# Exploring sesquiterpene lactones: structural diversity and antiviral therapeutic insights

**DOI:** 10.1039/d4ra08125k

**Published:** 2025-01-22

**Authors:** Yhiya Amen, Gehad Abdelwahab, Ahmed A. Heraiz, Mahmoud Sallam, Ahmed Othman

**Affiliations:** a Department of Pharmacognosy, Faculty of Pharmacy, Mansoura University Mansoura 35516 Egypt yhiaamen@mans.edu.eg; b Department of Pharmacognosy and Medicinal Plants, Faculty of Pharmacy (Boys), Al-Azhar University Cairo 11884 Egypt

## Abstract

Sesquiterpene lactones (SLs) are a structurally diverse group of secondary metabolites primarily produced by plants, particularly within the Asteraceae family. These compounds play significant roles in plant defense and have been extensively studied for their wide range of biological activities, including antiviral, antimicrobial, anti-inflammatory, and anticancer properties. This review focuses on the biosynthesis, structure–activity relationships, and biological activities of sesquiterpene lactones, with an emphasis on their antiviral potential. SLs exert their antiviral effects by targeting viral entry, replication, and other critical stages of the viral life cycle. Notable examples include guaianolides and germacranolides, which have demonstrated promising activity against viruses such as hepatitis C, influenza, and herpes simplex. This review also emphasizes the potential of sesquiterpene lactones as promising scaffolds for antiviral drug development, positioning these compounds as key candidates in combating viral infections.

## Abstract 2

Sesquiterpene lactones (SLs) are a structurally diverse group of secondary metabolites primarily produced by plants, particularly within the Asteraceae family.

## Graphical abstract

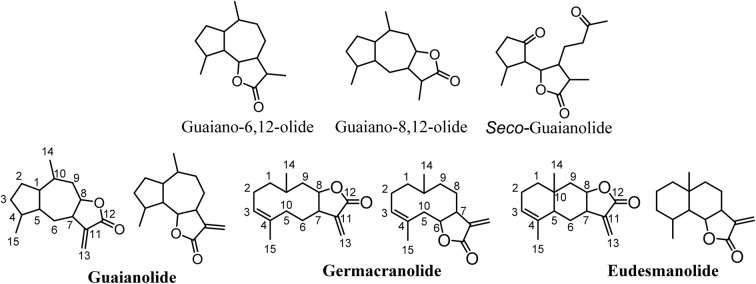

## 1. Introduction

Sesquiterpene lactones (SLs) constitute a large and structurally diverse secondary metabolites produced mainly by plants. These compounds have been observed in several plant families, such as Apiaceae, Magnoliaceae, Lauraceae, Cactaceae, Solanaceae, Araceae and Euphorbiaceae, with a special occurrence in the Asteraceae family.^1–3^

**Fig. 1:**
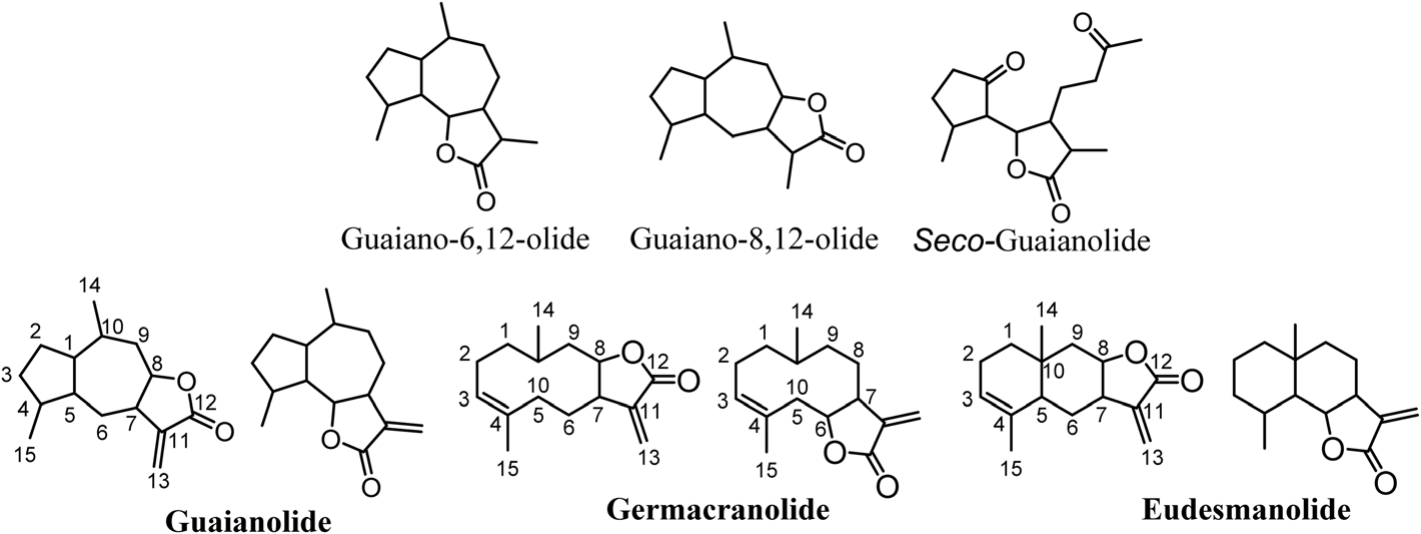
Main classes of sesquiterpene lactones.

SLs serve crucial functions in plant physiology, acting as deterrents, defense compounds (phytoalexins), allelochemicals, and agents that attract pollinators.^4^ They are terpenes with a 15-carbon skeleton (sesqui-) that contain an α,β-unsaturated carbonyl structure with an α-methylene-γ-lactone group. The stereochemistry of the lactone group can be *trans* or *cis* configuration; however, the *trans* configuration is the most common one.^3^ SLs are categorized based on their carbocyclic skeleton into pseudo-guaianolides, guaianolides (which encompass *seco*-guaianolides), germanocranolides, eudesmanolides, heliangolides, and hyptocretenolides, with guaianolides emerging as the common type (Fig. 1).

Concerning the structure activity relationships (SAR), the observed biological effect of SLs are attributed mainly to the α-methylene-γ-lactone group in their structures. Moreover, various studies have reported that the presence of alkylating centres, lipophilicity, and molecular geometry and electronic properties play a pivotal role in the biological properties of SLs.^3,5–7^ Of note, the existence of electrophilic domains associated with the moderate to high lipophilicity may be associated with their antimycobacterial efficacy, whereas the presence of a double bond in an exocyclic configuration relative to the cyclopentenone ring seemingly enhances their anti-inflammatory properties. Additionally, the presence of potentially reactive structural features, particularly α,β-unsaturated carbonyl moieties in the structure of SLs, has been correlated with their biological activities. Thus, many SLs exert multifaceted biological activities including anti-microbial, anti-fungal, anti-viral, anxiolytic, anti-tumor, anti-malarial, anti-trypanosomal, anti-diabetic, analgesic, and anti-inflammatory activities, as well as fragrances in the cosmetic industry.^4,8–10^ Notably, the mechanism of action of SLs has been correlated to α-methylene-γ-lactone and α,β-unsaturated cyclopentenone that act as alkylating agents on cellular proteins *via* Michael addition, particularly targeting their thiol groups. Consequently, they affect cellular processes such as gene expression, protein production, and metabolic pathways.^5,11^

Viruses are the most widespread living organisms on Earth, existing alongside bacteria, plants, and other animals, including humans. Most infectious diseases are caused by viruses. Aerosolized droplets through coughing and sneezing can spread viruses that cause tonsillitis, cold, bronchiolitis, influenza, pneumonia, and other respiratory tract infections. Since December 2019, a new coronavirus pneumonia pandemic, commonly known as COVID-19 and caused by SARS-CoV-2, has emerged from Wuhan City in China and posed a significant threat to global health. This virus infection led to around 4 million cases and more than 260 thousand deaths worldwide.^12^ Importantly, SLs are beneficial for human health as they can treat viral infections. To date, around 8000 SLs have been discovered; consequently, there has been a significant increase in the attention given to SLs owing to their diverse biological properties that are attributed to human health. This interest has led to many studies regarding the isolation of these compounds from natural sources, the development of semi-synthesis methodologies, and the evaluation of the pharmacological potential of SLs and their derivatives. Thus, sesquiterpene lactones are valuable scaffolds for drug discovery due to their interesting biological properties.

Based on the fact that further significant findings related to SLs are highly likely to emerge in the future, this review aims to highlight the antiviral SLs and the recent studies on their beneficial properties which could be a valuable route for the development of new antiviral drugs. Certainly, sesquiterpene lactones will remain a discovery pipeline in the field of natural products for many years.

## 2. Biosynthetic aspects of sesquiterpene lactones

**Fig. 2:**
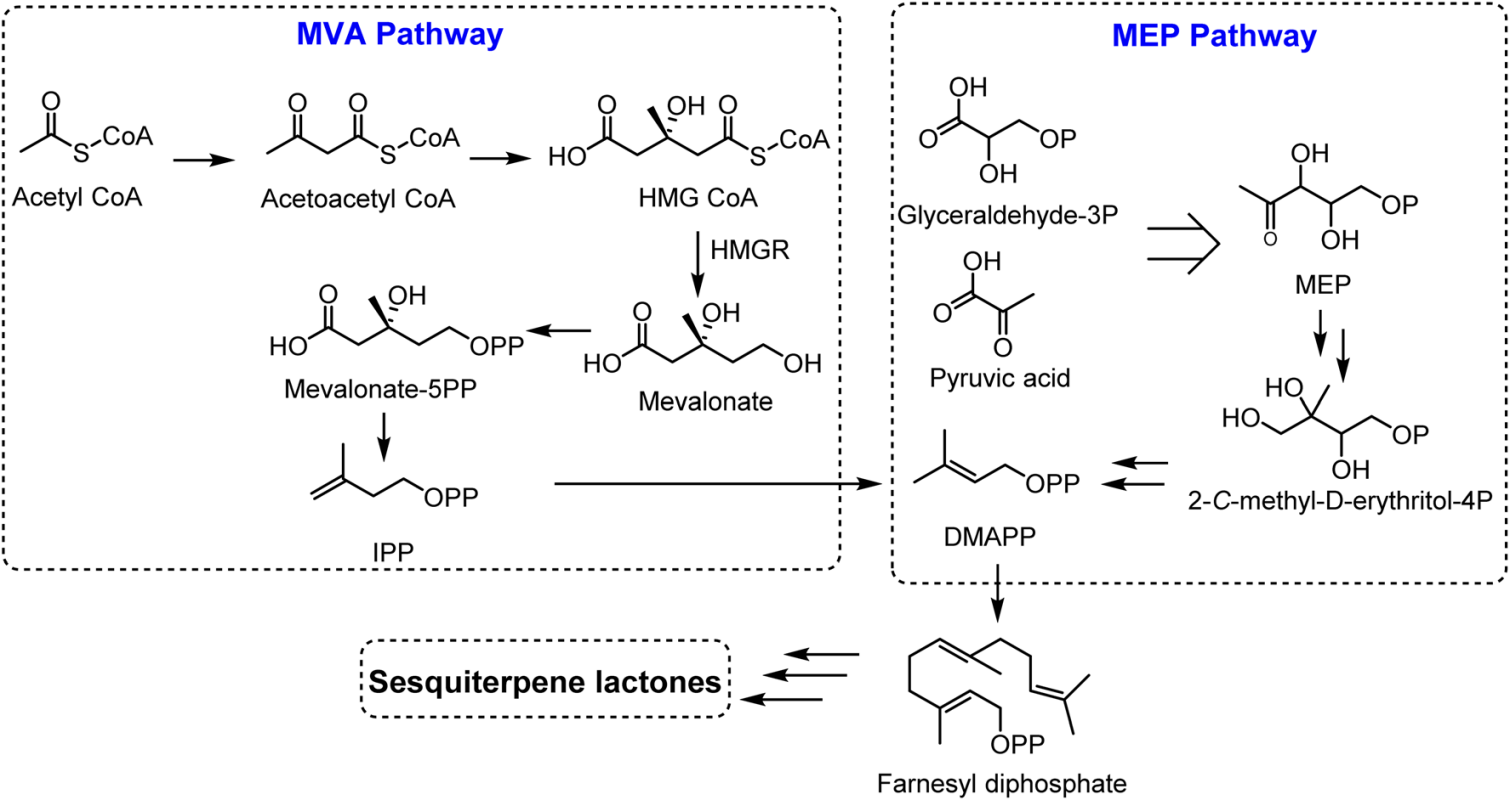
Biosynthetic pathway of sesquiterpene lactones.

Sesquiterpene lactones originated from isopentenyl diphosphate (IPP), which can be generated from two pathways; the mevalonate (MVA) pathway and the 2-*C*-methyl-d-erythritol 4-phosphate (MEP) pathway, localized in chloroplasts and cytosol.^4^ Glyceraldehyde-3-phosphate and pyruvic acid are converted to isopentenyl diphosphate (IPP) and dimethylallyl diphosphate (DMAPP) (5 : 1 ratio) through 7 enzymes in the MEP pathway. On the other hand, IPP is produced from acetyl-CoA in the MVA pathway in a sequence of 6 steps, followed by the conversion of IPP to its isomer, DMAPP, *via* isomerase enzyme (IPPI). Two molecules of IPPs and one molecule of DMAPP generate the precursor for all sesquiterpenoids, farnesyl diphosphate (FPP), *via* an action of farnesyl diphosphate synthase (FPS). Farnesyl diphosphate (FPP) is then transformed to sesquiterpenes by sesquiterpene synthases. Sesquiterpenes can be further modified by hydroxylation and oxidation reactions, mediated *via* cytochrome P450 enzymes (Fig. 2).

## 3. Antiviral activities of sesquiterpene lactones

**Fig. 3:**
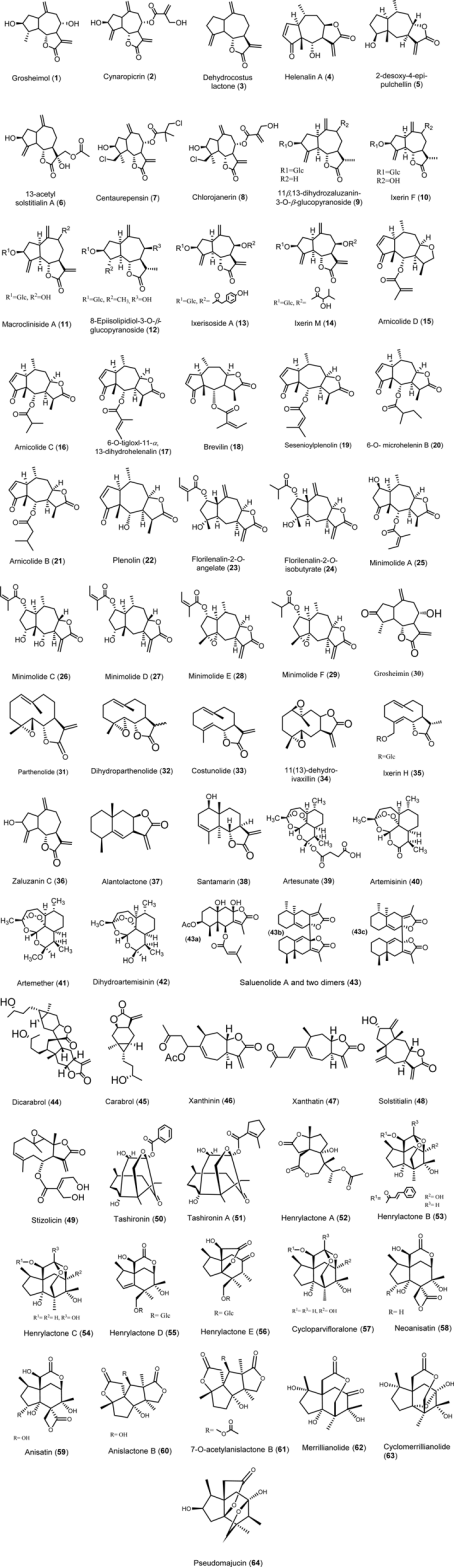
Structure of sesquiterpene lactones (1–64).

Sesquiterpene lactones (SLs) exhibit significant antiviral activities, categorized into several structural classes including guaianolides, germacranolides, eudesmanolides, xanthanolides, and others. Guaianolides such as grosheimol and cynaropicrin show potent inhibition of multiple HCV genotypes, while compounds like dehydrocostus lactone effectively target HBV. Germacranolides, especially parthenolide, demonstrate a broad spectrum of antiviral efficacy against HCV, HSV-1, and SARS-CoV-2. Eudesmanolides like alantolactone exhibit activity against HCV, and xanthanolides such as xanthatin show antiviral effects against various viruses. These SLs display their activities through diverse mechanisms, including inhibition of viral entry, RNA replication, and protein expression, highlighting their potential as antiviral agents across several viral families. The details of various sesquiterpene lactones (STLs) with potential antiviral activity are illustrated in Fig. 3 and elaborated upon in the following sections. A summary of their activity profiles is provided in Table 1.

**Table 1:** Antiviral activities of reported sesquiterpene lactones^a^

#	Compounds	Class	Source	Virus	IC_50_ or EC_50_ or CPE	Cell lines	Ref.
1	Grosheimol	Guaianolide	*Cynara scolymus* L	HCV (Luc-Jc1)	EC_50_ = 1.03 μM	Huh7/Scr	13
				Genotypes			
				1a (TN)	EC_50_ = 2.7 μM		
				1b (J4)	EC_50_ = 4.5 μM		
				2b (J8)	EC_50_ = 7.23 μM		
				3a (S52)	EC_50_ = 7.2 μM		
				4a (ED43)	EC_50_ = 8.7 μM		
				5a (SA13)	EC_50_ = 13.9 μM		
				6a (HK6a)	EC_50_ = 6.3 μM		
				7a (QC69)	EC_50_ = 5.7 μM		
2	Cynaropicrin	Guaianolide	*Cynara scolymus* L	HCV (Luc-Jc1)	EC_50_ = 1.27 μM	Huh7/Scr	13
				Genotypes			
				1a (TN)	EC_50_ = 0.4 μM		
				1b (J4)	EC_50_ = 1.1 μM		
				2b (J8)	EC_50_ = 0.7 μM		
				3a (S52)	EC_50_ = 0.7 μM		
				4a (ED43)	EC_50_ = 0.7 μM		
				5a (SA13)	EC_50_ = 0.8 μM		
				6a (HK6a)	EC_50_ = 0.7 μM		
				7a (QC69)	EC_50_ = 1.4 μM		
3	Dehydrocostus lactone	Guaianolide	Roots of *Saussurea lappa* Clarks	HBsAg	IC_50_ = 2 μM	Hep3B	14
				Hepatitis C virus (HCV)	EC_50_ = 3.08 ± 0.60 μM	Ava5-EG (Δ4AB) SEAP cells (sub-genomic replicon HCV *in vitro* inhibitory assay)	15–17
				HSV-1	% antiviral activity = 85.35 ± 9.2	Vero cells	18
				HAV	% antiviral activity = 23.02 ± 7.43 μg mL^−1^	Vero cells	18
4	Helenalin A	Pseudoguaianolide	Whole plants of *Anaphalis morrisonicola*	Hepatitis C virus HCV	EC_50_ ≤ 3 μM	Huh-7	15, 16, 19 and 20
					EC_50_ = 1.25 ± 0.35 μM	Ava5-EG (Δ4AB) SEAP cells (sub-genomic replicon HCV *in vitro* inhibition assay)	
				Influenza virus A/Puerto Rico/8/34 H1N1 (PR8)	Not determined	(CPE) reduction assay	21
5	2-Desoxy-4-*epi*-pulchellin	Guaianolide	Whole plants of *Carpesium abrotanoides* L	H1N1 H3N2	IC_50_ = 29.3 μM	Madin–Darby canine kidney (MDCK) cells	22
					IC_50_ = 47.3 μM		
6	13-Acetyl-solstitialin	Guaianolide	Aerial parts of *Centaurea solstitialis* L. ssp. *solstitialis*	Herpes simplex type-1	CPE = 16 μg mL^−1^	Vero cells	23 and 24
				Parainfluenza virus	Not active	Vero cells	23 and 24
7	Centaurepensin	Guaianolide	Aerial parts of *Centaurea solstitialis* L. ssp. *Solstitialis*	Herpes simplex type-1	CPE = 2 μg mL^−1^	Vero cells	23
				Parainfluenza virus	Not active	Vero cells	23
8	Chlorojanerin	Guaianolide	Aerial parts of *Centaurea solstitialis* L. ssp. *solstitialis*	Herpes simplex type-1	CPE = 2 μg mL^−1^	Vero cells	23
				Parainfluenza virus	Not active	Vero cells	23
9	11β,13-Dihydrozaluzanin-3-*O*-β-glucopyranoside	Guaianolide	Roots of *Ixeris dentata*		Antiviral activity index (%)	Vero cells	15, 16, 19 and 20
				Coxsackievirus B (CVB3)	2.15 ± 1.11		
				Human enterovirus 71 (EV71)	38.00 ± 0.50		
10	Ixerin F	Guaianolide	Roots of *Ixeris dentata*		Antiviral activity index (%)	Vero cells	25
				Coxsackievirus B (CVB3)	1.85 ± 1.10		
				Human enterovirus 71 (EV71)	83.51 ± 1.73		
11	Macrocliniside A	Guaianolide	Roots of *Ixeris dentata*		Antiviral activity index (%)	Vero cells	25
				Coxsackievirus B (CVB3)	5.45 ± 1.19		
				Human enterovirus 71 (EV71)	28.02 ± 0.32		
12	8-Epiisolipidiol-3-*O*-β-glucopyranoside	Guaianolide	Roots of *Ixeris dentata*		Antiviral activity index (%)	Vero cells	25
				Coxsackievirus B (CVB3)	15.81 ± 3.60		
				Human enterovirus 71 (EV71)	14.77 ± 1.05		
13	Ixerisoside A	Guaianolide	Roots of *Ixeris dentata*		Antiviral activity index (%)	Vero cells	25
				Coxsackievirus B (CVB3)	41.87 ± 2.14		
				Human enterovirus 71 (EV71)	−8.89 ± 0.98		
14	Ixerin M	Guaianolide	Roots of *Ixeris dentata*		Antiviral activity index (%)	Vero cells	25
				Coxsackievirus B (CVB3)	45.53 ± 2.24		
				Human enterovirus 71 (EV71)	−3.03 ± 0.82		
15	Arnicolide D	Pseudoguaianolide	Whole herbs of *C. Minima*	Influenza virus A/Puerto Rico/8/34 H1N1 (PR8)	IC_50_ = 11.6 ± 1.3 μM (SI = 4.3)	MDCK cells	21
						(CPE) reduction assay	
16	Arnicolide C	Pseudoguaianolide	Whole herbs of *C. Minima*	Influenza virus A/Puerto Rico/8/34 H1N1 (PR8)	IC_50_ = 4.9 ± 0.8 μM (SI = 9.2)	MDCK cells	21
						(CPE) reduction assay	
17	6-*O*-Tigloxl-11α, 13-dihydrohelenalin	Pseudoguaianolide	Whole herbs of *C. Minima*	Influenza virus A/Puerto Rico/8/34 H1N1 (PR8)	IC_50_ = 2.8 ± 0.8 μM (SI = 11.8)	MDCK cells	21
						(CPE) reduction assay	
18	Brevilin A	Pseudoguaianolide	Whole herbs of *C. Minima*	Influenza virus A/Puerto Rico/8/34 H1N1 (PR8)	IC_50_ = 1.8 ± 0.6 μM (SI = 13.5)	MDCK cells	21
						(CPE) reduction assay	
				Influenza A/PR/8/34 (H1N1)	EC_50_ = 2.96 ± 1.10 μM (SI = 8)	MDCK cells	26
				Influenza A/FM/1/47 H1N1	EC_50_ = 1.60 ± 1.14 μM (SI = 14)		
				Influenza A/Hong Kong/498/97 H3N2	EC_50_ = 3.28 ± 1.09 μM (SI = 7)		
				Influenza A/chicken/Guangdong/1996 H9N2	EC_50_ = 2.07 ± 1.12 μM (SI = 11)		
19	Sesenioylplenolin	Pseudoguaianolide	Whole herbs of *C. Minima*	Influenza virus A/Puerto Rico/8/34 H1N1 (PR8)	IC_50_ = 3.5 ± 1.6 μM (SI = 7.5)	MDCK cells	21
						(CPE) reduction assay	
20	6-*O*-Microhelenin B	Pseudoguaianolide	Whole herbs of *C. Minima*	Influenza virus A/Puerto Rico/8/34 H1N1 (PR8)	IC_50_ = 5.6 ± 0.8 μM (SI = 6.9)	MDCK cells	21
						(CPE) reduction assay	
21	Arnicolide B	Pseudoguaianolide	Whole herbs of *C. Minima*	Influenza virus A/Puerto Rico/8/34 H1N1 (PR8)	IC_50_ = 2.1 ± 0.5 μM (SI = 9.4)	MDCK cells	21
						(CPE) reduction assay	
22	Plenolin	Pseudoguaianolide	Whole herbs of *C. Minima*	Influenza virus A/Puerto Rico/8/34 H1N1 (PR8)	IC_50_ = 4.6 ± 1.1 μM (SI = 8.1)	MDCK cells	21
						(CPE) reduction assay	
23	Florilenalin-2-*O*-angelate	Guaianolide	Whole herbs of *C. Minima*	Influenza virus A/Puerto Rico/8/34 H1N1 (PR8)	IC_50_ ≤ 25 μM	MDCK cells	21
						(CPE) reduction assay	
24	Florilenalin-2-*O*-isobutyrate	Guaianolide	Whole herbs of *C. Minima*	Influenza virus A/Puerto Rico/8/34 H1N1 (PR8)	IC_50_ ≤ 25 μM	MDCK cells	21
						(CPE) reduction assay	
25	Minimolide A	Pseudoguaianolide	Whole herbs of *C. Minima*	Influenza virus A/Puerto Rico/8/34 H1N1 (PR8)	IC_50_ ≤ 50 μM	MDCK cells	21
						(CPE) reduction assay	
26	Minimolide C	Pseudoguaianolide	Whole herbs of *C. Minima*	Influenza virus A/Puerto Rico/8/34 H1N1 (PR8)	IC_50_ ≤ 50 μM	MDCK cells	21
						(CPE) reduction assay	
27	Minimolide D	Pseudoguaianolide	Whole herbs of *C. Minima*	Influenza virus A/Puerto Rico/8/34 H1N1 (PR8)	IC_50_ ≤ 50 μM	MDCK cells	21
						(CPE) reduction assay	
28	Minimolide E	Guaianolide	Whole herbs of *C. Minima*	Influenza virus A/Puerto Rico/8/34 H1N1 (PR8)	IC_50_ ≤ 50 μM	MDCK cells	21
						(CPE) reduction assay	
29	Minimolide F	Guaianolide	Whole herbs of *C. Minima*	Influenza virus A/Puerto Rico/8/34 H1N1 (PR8)	IC_50_ ≤ 50 μM	MDCK cells	21
						(CPE) reduction assay	
30	Grosheimin	Guaianolide	*Chartolepis intermedia* Boiss	Influenza	N/A	*In vitro*	27
31	Parthenolide	Germacranolide	*Tanacetum parthenium* L. aerial parts	HBV and HCV	N/A	*In vitro*	27
			Aerial parts of *Tanacetum vulgare* L. and *Tanacetum parthenium* L. (feverfew)	(HCV)	EC_50_ ≤ 3 μM	Huh-7	20
				HSV-1 (KOS strain)	MTT method	Vero cells	28 and 29
					EC_50_ = 0.3 ± 0.01 μg mL^−1^		
					Plaque reduction method		
					EC_50_ = 2.3 ± 0.02 μg mL^−1^		
			*Tanacetum parthenium* L. aerial parts	HSV-1 (AR-29 strain)	Plaque reduction method	Vero cells	29
					EC_50_ = 1.8 ± 0.07 μg mL^−1^		
				SARS-CoV-2	IC_50_ = 132.5 μM	HeLa cells transfected with Flag-tagged SARS-CoV-2 PL^pro^	30
				Hepatitis C virus (HCV)	EC_50_ = 2.21 ± 0.15 μM	Ava5-EG (Δ4AB) SEAP cells (sub-genomic replicon HCV *in vitro* inhibitory assay)	15 and 17
32	Dihydroparthenolide	Germacranolide	*Michelia lanuginosa* Wall. trunk bark *Ambrosia artemisiifolia* L. (common ragweed)	HCV	EC_50_ ≤ 3 μM	Huh-7	20 and 31
33	Costunolide	Germacranolide	Roots of *Saussurea lappa* Clarks	HBsAg	IC_50_ = 1 μM	Hep3B	14
				HCV	EC_50_ ≤ 3 μM	Huh-7	20
				HSV-1	92.91 ± 2.47% antiviral activity	Vero cells	18
				HAV	42.71 ± 10.47% antiviral activity	Vero cells	18
				Hepatitis C virus (HCV)	EC_50_ = 2.69 ± 0.57 μM	Ava5-EG (Δ4AB) SEAP cells (sub-genomic replicon HCV *in vitro* inhibitory assay)	15, 17 and 32
34	11(13)-Dehydroivaxillin	Germacranolide	Whole plant of *Carpesium abrotanoides* L. (Asteraceae)	H1N1 H3N2	IC_50_ = 10.8 μM	Madin–Darby canine kidney (MDCK) cells	22
					IC_50_ = 11.6 μM		
35	Ixerin H	Germacranolide	Roots of *Ixeris dentata*	Antiviral activity index (%)		Vero cells	25
				Coxsackievirus B (CVB3)	33.73 ± 1.93%		
				Human enterovirus 71 (EV71)	61.70 ± 1.12%		
36	Zaluzanin C	Germacranolide	*Saussurea lappa*	HSV-1	Inhibitory activity% = 79.93 ± 7.17 μg mL^−1^	Vero cells	18
				HAV	Inhibitory activity% = 53.7 ± 10.05 μg mL^−1^		
37	Alantolactone	Eudesmanolide	Roots of *Inula Helenium*	HCV	EC_50_ ≤ 3 μM	Huh-7	20 and 33
				Hepatitis C virus (HCV)	EC_50_ = 2.03 ± 0.15 μM	Ava5-EG (Δ4AB) SEAP cells (sub-genomic replicon HCV *in vitro* inhibitory assay)	15–17
38	Santamarin	Eudesmanolide	*Saussurea lappa*	HSV-1	Inhibitory activity% = 92.91 ± 2.47 μg mL^−1^	Vero cells	18
				HAV	Inhibitory activity% = 42.71 ± 10.47 μg mL^−1^		
39	Artesunate	Eudesmanolide (endoperoxide type)	*Artemisia annua* [Qinghao (the blue-green herb)]	Hepatitis B virus (HBV)		HepG2 2.2.15	15 and 34
				HBsAg	IC_50_ = 2.3 μM		
				HBV-DNA	IC_50_ = 0.5 μM		
			Water-soluble, semi-synthetic derivative of artemisinin	(BVDV-strain Pe515)	EC_50_ = 0.07 μg mL^−1^	(MDBK) cells	35
				Human cytomegalovirus (HCMV)	Significant reduction in viral load at day 7, with a virus half-life of 0.9–1.9 days	12 year patient with (HCMV) infection	36
40	Artemisinin	Eudesmanolide (endoperoxide type)	*Artemisia annua* [Qinghao (the blue-green herb)]	Hepatitis B virus (HBV)		HepG2 2.2.15	15 and 34
				HBsAg	IC_50_ = 55 μM		
				HBV-DNA	IC_50_ ≥ 100 μM		
				Bovine viral diarrhoea virus (BVDV-strain Pe515)	EC_50_ = 0.4 μg mL^−1^	Bovine kidney (MDBK) cells	35
			Aerial parts of *Artemisia annua* (sweet wormwood) (Asteraceae)	Hepatitis C virus (HCV)	EC_50_ ≥ 10 μM	Ava5-EG (Δ4AB) SEAP cells (sub genomic replicon HCV *in vitro* inhibtory assay)	15–17
41	Artemether	Eudesmanolide (endoperoxide type)	Semi-synthetic derivative of artemisinin	(BVDV-strain Pe515)	EC_50_ = 0.3 μg mL^−1^	(MDBK) cells	35
42	Dihydroartemisinin	Eudesmanolide (endoperoxide type)	*Artemisia annua* L. leaves	(BVDV-strain Pe515)	EC_50_ = 0.05 μg mL^−1^	(MDBK) cells	35
43	Saluenolide A and two dimers	Eremophilanolide	Whole plants of *Senecio tsoongianus*	Hepatitis B		HepG2.2.15	37
				HBsAg	(43a)		
					IC_50_ = 78.7 ± 3.9 mM		
					(43b)		
					IC_50_ = 89.3 ± 10.6 mM		
					(43c)		
					IC_50_ = 101.5 ± 9.3 mM		
				HBeAg	(43a)		
					IC_50_ = 93.5 ± 11.5 mM		
					(43b)		
					IC_50_ = 99.4 ± 7.4 mM		
					(43c)		
					IC_50_ = 87.2 ± 9.5 mM		
				Extracellular HBV DNA	(43a)		
					IC_50_ = 42.3 ± 7.6 mM		
					(43b)		
					IC_50_ = 38.0 ± 4.5 mM		
					(43c)		
					IC_50_ = 79.6 ± 6.8 mM		
				Intracellular HBV DNA	(43a)		
					(43b)		
					(43c)		
					IC_50_ < 250 (no inhibition)		
				HBsAg	(43a)		
					IC_50_ = 78.7 ± 3.9 mM		
					(43b)		
					IC_50_ = 89.3 ± 10.6 mM		
					(43c)		
					IC_50_ = 101.5 ± 9.3 mM		
44	Dicarabrol	Carabrane sesquiterpenoid dimer	Whole plant of *Carpesium abrotanoides* L	H1N1	IC_50_ = 15.9 μM	Madin–Darby canine kidney (MDCK) cells	22
				H3N2	IC_50_ = 30.0 μM		
45	Carabrol	Carabrane sesquiterpenoid	Whole plant of *Carpesium abrotanoides* L	H1N1	IC_50_ = 45.5 μM	Madin–Darby canine kidney (MDCK) cells	22
				H3N2	IC_50_ ≥ 100 μM		
46	Xanthinin	Xanthanolide	Aerial fresh young parts of *Xanthium spinosum* L	Not tested	N/A	N/A	38
47	Xanthatin	Xanthanolide	Aerial fresh young parts of *Xanthium spinosum* L. (Asteraceae)	Para-influenza-3 virus	EC_50_ ≥ 20 μM	Human embryonic lung (HEL) fibroblasts, African green monkey cells (VERO), human epithelial cells (HeLa) or in Crandell–Rees feline kidney cells (CRFK)	38
				Reovirus-1	EC_50_ ≥ 20 μM		
				Sindbis virus	EC_50_ ≥ 20 μM		
				Coxsackie virus B4	EC_50_ ≥ 20 μM		
				Punta Toro virus	EC_50_ ≥ 20 μM		
48	Solstitialin	Xanthanolide	Aerial fresh young parts of *Xanthium spinosum* L	Not tested	N/A	N/A	38
49	Stizolicin	Xanthanolide	Aerial fresh young parts of *Xanthium spinosum* L	Not tested	N/A	N/A	38
50	Tashironin	Unusual SL	Stems and roots of *Illicium henryi*	Hepatitis B virus (HBV)		HepG2.2.15	39
				HBsAg	IC_50_ = 0.48 mM (SI = 6.3)		
				HBeAg	IC_50_ = 0.15 mM (SI = 20.1)		
			Roots of *Illicium verum* Hook. F	Human immunodeficiency virus (HIV)	EC_50_ = 44.0 μM (SI = 5.4)	C8166 cells	40
51	Tashironin A	Unusual SL	Stems and roots of *Illicium henryi*	Hepatitis B virus (HBV)		HepG2.2.15	39
				HBsAg	IC_50_ = 0.49 mM (SI = 2.1)		
				HBeAg	IC_50_ = 0.15 mM (SI = 6.7)		
			Roots of *Illicium verum* Hook. F	Human immunodeficiency virus (HIV)	EC_50_ = 41.8 μM (SI = 6.2)	C8166 cells	40
52	Henrylactone A	Unusual SLs	Stems and roots of *Illicium henryi*	Hepatitis B virus (HBV)		HepG2.2.15	39
				HBsAg	IC_50_ = 1.85 mM (SI = 1.1)		
				HBeAg	IC_50_ = 1.70 mM (SI = 1.2)		
53	Henrylactone B	Unusual SLs	Stems and roots of *Illicium henryi*	Hepatitis B virus (HBV)		HepG2.2.15	39
				HBsAg	IC_50_ = 0.098 mM (SI = 2.4)		
				HBeAg	IC_50_ = 0.24 mM (SI = 1.0)		
54	Henrylactone C	Unusual SL	Stems and roots of *Illicium henryi*	Hepatitis B virus (HBV)		HepG2.2.15	39
				HBsAg	IC_50_ = 1.30 mM (SI = 1.9)		
				HBeAg	IC_50_ = 1.80 mM (SI = 1.4)		
55	Henrylactone D	Unusual SL	Stems and roots of *Illicium henryi*	Hepatitis B virus (HBV)		HepG2.2.15	39
				HBsAsg	IC_50_ = 0.63 mM (SI = 1.2)		
				HBeAg	IC_50_ = 0.63 mM (SI = 1.2)		
56	Henrylactone E	Unusual SL	Stems and roots of *Illicium henryi*	Hepatitis B virus (HBV)		HepG2.2.15	39
				HBsAg	IC_50_ = 1.09 mM (SI ≥ 2.7)		
				HBeAg	IC_50_ = 2.91 mM (SI ≥ 1.0)		
57	Cycloparvifloralone	Unusual SL	Stems and roots of *Illicium henryi*	Hepatitis B virus (HBV)		HepG2.2.15	39
				HBsAg	IC_50_ ≥ 5.50 mM		
				HBeAg	IC_50_ ≥ 5.50 mM		
58	Neoanisatin	Unusual SL	Stems and roots of *Illicium henryi*	Hepatitis B virus (HBV)		HepG2.2.15	39
				HBsAg	IC_50_ ≥ 2.91 mM		
				HBeAg	IC_50_ ≥ 2.91 mM		
59	Anisatin	Unusual SL	Stems and roots of *Illicium henryi*	Hepatitis B virus (HBV)		HepG2.2.15	39
				HBsAg	IC_50_ ≥ 3.26 mM		
				HBeAg	IC_50_ ≥ 3.26 mM		
60	Anislactone B	Unusual SL	Stems and roots of *Illicium henryi*	Hepatitis B virus (HBV)		HepG2.2.15	39
				HBsAg	IC_50_ = 1.45 mM (SI = 1.1)		
				HBeAg	IC_50_ = 0.91 mM (SI = 1.8)		
61	7-*O*-Acetylanislactone B	Unusual SL	Stems and roots of *Illicium henryi*	Hepatitis B virus (HBV)		HepG2.2.15	39
				HBsAg	IC_50_ ≥ 3.90 mM		
				HBeAg	IC_50_ ≥ 3.90 mM		
62	Merrillianolide	Unusual SL	Stems and roots of *Illicium henryi*	Hepatitis B virus (HBV)		HepG2.2.15	39
				HBsAg	IC_50_ = 1.74 mM (SI ≥ 2.1)		
				HBeAg	IC_50_ ≥ 3.59 mM		
63	Cyclomerrillianolide	Unusual SL	Stems and roots of *Illicium henryi*	Hepatitis B virus (HBV)		HepG2.2.15	39
				HBsAg	IC_50_ = 3.25 mM (SI ≥ 1.0)		
				HBeAg	IC_50_ ≥ 3.41 mM		
64	Pseudomajucin	Unusual SL	Stems and roots of *Illicium henryi*	Hepatitis B virus (HBV)		HepG2.2.15	39
				HBsAg	IC_50_ ≥ 4.40 mM		
				HBeAg	IC_50_ ≥ 4.40 mM		

a CPE: cytopathogenic effect, N/A: not available.

### 3.1. Antiviral activities of guaianolide SLs

Guaianolide constitutes a wide range of sesquiterpene lactones with diverse biological activities. This kind of sesquiterpene lactones is based on a 5,7,5-ring system that could be guaian-6,12-olide, guaian-8,12-olide, or *seco*-guaianolides that characterized by the cleavage of single C–C bond in one of the rings. Such kind of sesquiterpene lactones display diverse pharmacological activities, including antiviral, antimicrobial, antitumor, and anti-inflammatory activity.^1^

Grosheimol (1) and cynaropicrin (2) are guaianolide sesquiterpene lactone derivatives formed basically from perhydro-azulen attached to the lactone ring. Grosheimol contains two *exo*-olefins while cynaropicrin contains four *exo*-olefins as shown in Fig. 3. They were first reported from artichoke; *Cynara scolymus* L.^13^ Grosheimol (1) and cynaropicrin (2) are strong inhibitors for HCV infection with a broad spectrum activity toward multiple HCV genotypes (1a, 1b, 2b, 3a, 4a, 5a, 6a, and 7a). Grosheimol and cynaropicrin showed significant activity against Luc-Jc1 virus with EC_50_ of 1.03 and 1.27 μM, respectively. Huh7/Scr cells (“donor cells”) were infected with the Jc1 virus at a multiplicity of infection (MOI) of ≥5 TCID_50_/cell.

Grosheimol and cynaropicrin promote antiviral activity against HCV at the entry level into target cells rather than replication. Additionally, the early entry and virus binding phases of the HCV life cycle were inhibited by grosheimol and cynaropicrin. Thus, both compounds were suggested to act directly toward all HCV genotypes *via* their effects on virus particles and inhibiting virus–receptor interactions; subsequently, grosheimol and cynaropicrin are promising pan-genotypic anti-HCV novel natural products.^13^

Dehydrocostus lactone (3), isolated from the roots of the Chinese medicinal herb *Saussurea lappa*, exhibited a potent suppressive effect on hepatitis B surface antigen (HBsAg) in human hepatoma Hep3B cells, with an IC_50_ value of 2 μM.^14^ It also demonstrated anti-HCV activity, with an EC_50_ of 3.08 ± 0.60 μM, potentially due to the presence of an *exo*-methylene lactone functional group. Alongside three other eudesmanolides from *Saussurea lappa* roots, dehydrocostus lactone (3) showed notable antiviral effects against herpes simplex virus type 1 (HSV-1) and hepatitis A virus (HAV), with activity percentages of 85.35 ± 9.2% and 23.02 ± 7.43%, respectively, compared to the standard acyclovir. Additionally, molecular docking simulations suggested that its antiviral mechanism may involve binding to HSV-1 DNA polymerase and HAV 3C proteinase enzymes.^18^

Another antiviral guaianolide has been isolated from chloroform extract of the whole plant *Anaphalis morrisonicola*, namely helenalin A (4) in 1977,^19^ and later in 2004, helenalin A has been reported to exert anti-HCV through inhibition of RNA replication with EC_50_ less than 3 μM.^20^

2-Desoxy-4-*epi*-pulchellin (5) has been isolated from the whole plants of *Carpesium abrotanoides* L, a biennial herb belonging to the Asteraceae family which is widely distributed in China and Korea.^41^ This compound has showed anti-influenza A H1N1 and H3N2 viruses' activity with IC_50_ values of activity 29.3 and 47.3 μM.^22^

Three sesquiterpene lactones, 13-acetyl solstitialin A (6), centaurepensin or chlorohyssopifolin A (7), and chlorojanerin (8), were isolated from the chloroform extract of the aerial parts of *Centaurea solstitialis* L. ssp. solstitialis (Asteraceae). The antiviral activity of 13-acetyl solstitialin A (6) (16–0.00006 μg mL^−1^) toward the DNA virus HSV-1 was as potent as reference drug, acyclovir with minimum nontoxic concentration (MNTC) of 16 μg mL^−1^. Centaurepensin (7) and chlorojanerin (8) with MNTC of 2 μg mL^−1^ were active against the DNA virus HSV-1 and were less active than 13-acetyl solstitialin A. All three sesquiterpene lactones were totally inactive against RNA virus (PIV).^23,24^ The α-methylene-γ-lactone moiety, which acts as a Michael acceptor, is a key structural feature in sesquiterpene lactones that contributes to their biological activity. Additionally, the study found a positive correlation between antiviral activity and the presence of multiple chlorinated substituents. For instance, centaurepensin contains two chlorinated groups, while chlorojanerin has one. However, the most active compound, 13-acetyl solstitialin A, lacks both chlorinated groups and the α-methylene-γ-lactone moiety. This observation suggests that these two structural features may not be essential for achieving significant antiviral activity.^23^

Six guaianolide sesquiterpene lactone glucosides (9–14) with one germacronolide were isolated from the roots of *Ixeris dentata*, namely, 11β,13-dihydrozaluzanin-3-*O*-β-glucopyranoside (9), ixerin F (10), macrocliniside A (11), 8-epiisolipidiol-3-*O*-β-glucopyranoside (12), ixerisoside A (13), and ixerin M (14) were evaluated in terms of their antiviral activities against coxsackievirus B3 (CVB3) and human enterovirus 71 (EV71). For the antiviral activities, guaianolides with an ester group at C-8; compounds 13 and 14 showed the most potent activities against CVB3 with an antiviral activity index of 41.87 ± 2.14% and 45.53 ± 2.24%, respectively.^25^

The supercritical fluid extract (SFE) of *Centipeda minima* possessed a good antiviral activity against influenza virus A/Puerto Rico/8/34 H1N1 (PR8) *in vitro*. Bioassay-guided isolation led to the isolation of seven pseudoguaianolides (15–21). These, as well as nine other sesquiterpene lactones (4, 22–29) previously isolated from this herb, were all tested for their anti-PR8 activity using both the cytopathogenic effect (CPE) reduction and cell counting kit 8 (CCK8) assays. As a result, eight pseudoguaianolides (15–22) possessing an α,β-unsaturated cyclopentenone moiety showed antiviral activity against PR8 with different extents. Of the active compounds, brevilin A (18) exhibited the strongest anti-PR8 activity, with an IC_50_ value much lower than that of the positive control ribavirin. Mechanistic study revealed that brevilin A affected the intracellular replication of PR8 *via* downregulating the expression of viral M2 protein. All these results suggest the potential application of the pseudoguaianolides containing an α,β-unsaturated cyclopentenone moiety (*e.g.* brevilin A) in the treatment of influenza virus infection.^21^

Zhang *et al.* explored the antiviral properties and mechanisms of action of brevilin A (18) against various influenza A virus (IAV) subtypes.^26^ Their findings demonstrated that brevilin A effectively inhibited the infection of influenza A/PR/8/34 (H1N1) *in vitro*, reducing the replication of influenza A H1N1, H3N2, and H9N2 strains. Notably, the antiviral effect of brevilin A was evident as early as 4 to 8 hours post-infection. The study revealed that brevilin A interfered with viral replication by targeting three key aspects: the synthesis of viral RNA (vRNA), the expression of viral mRNA and associated proteins encoded by the M and NS segments, and the nuclear export of viral ribonucleoproteins (vRNPs). In addition, the *in vivo* efficacy of brevilin A was confirmed in mice, where treatment (25 mg kg^−1^) resulted in a delayed time-to-death, with 50% of treated animals surviving up to 14 days post-infection compared to the control group. Based on these findings, the authors proposed that sesquiterpene lactones (SLTs) with structural similarities to brevilin A hold promise as potential anti-influenza agents. Collectively, these results highlight brevilin A and related SLTs as encouraging candidates for further development in influenza therapy.

Grosheimin (30), a compound isolated from the aerial parts of *Chartolepis intermedia* Boiss., has demonstrated significant antiviral activity, particularly against influenza viruses. Its water-soluble form is currently being recommended for further pre-clinical and clinical trials due to its potential as a novel antiviral agent. The unique structure of grosheimin facilitates effective interactions with viral components, contributing to its antiviral efficacy. Additionally, several chemical modifications of grosheimin have been investigated to enhance its biological activity, leading to the development of derivatives with improved antiviral properties. These findings underscore grosheimin's potential as a promising candidate for antiviral drug development.^27^

### 3.2. Antiviral activities of germacranolide SLs

Germacranolide is the largest group of sesquiterpene lactones (SLs) possessing a 10,5-ring structure and reported in several plant families. They serve as key precursors for the generation of various SLs with different polycyclic skeletons, such as guaianolides, eudesmanolides, and other sesquiterpenenoids.^42^

In their chemical investigation of *Tanacetum vulgare* L., Onozato and colleagues identified a germacranolide-type sesquiterpene lactone known as parthenolide (31)^28^ isolated from *Tanacetum parthenium* L., commonly referred to as feverfew. Parthenolide has garnered considerable attention due to its diverse biological activities, which include anti-inflammatory, antitumor, antibacterial, antifungal, and notably, antiviral properties. Over 500 publications have documented its effectiveness as a biologically active substance, highlighting its potential therapeutic applications. Notably, research has shown that parthenolide exhibits antiviral activity against hepatitis B and C viruses, positioning it as a promising candidate for treating viral infections. Key structural features, such as the α-methylene-γ-lactone ring, contribute to its pharmacological efficacy, including its antiviral effects.^27,43^

Parthenolide (31) also demonstrated significant antiviral activity against HSV-1 by impairing cell viability and reducing the production of viral particles, underscoring its potential as a therapeutic agent for herpes simplex virus infections. Both HSV-1 strains tested (KOS and AR-29) exhibited sensitivity to parthenolide, maintaining its antiviral activity even against the acyclovir-resistant AR-29 strain. Importantly, parthenolide's antiviral effects were observed only after the virus had penetrated the host cell, indicating that its action occurs post-infection rather than during the initial stages of viral entry. Treatment with parthenolide resulted in decreased expression of essential viral proteins such as gB, gD, and ICP0, which are crucial for HSV-1 particle production, with an EC_50_ of 0.3 μg mL^−1^. This reduction implies that parthenolide interferes with the virus's ability to replicate and produce new viral particles.^20,29^

Moreover, parthenolide (31) was found to inhibit the deISGylation activity of SARS-CoV-2 papain-like protease (PL^pro^) without affecting its deubiquitinating activity, indicating a selective inhibition mechanism. Molecular docking studies revealed that parthenolide covalently binds to critical cysteine residues (Cys-191 and Cys-194) of PL^pro^, which are essential for its enzymatic function. Consequently, parthenolide could serve as a foundation for developing new PL^pro^ inhibitors, which may be particularly beneficial for treating COVID-19, especially given the urgent need for effective antiviral agents.^30^

Additionally, parthenolide (31), along with dihydroparthenolide (32) and costunolide (33), exhibited potent dose-dependent inhibitory activities against the RNA replication of HCV, with EC_50_ values below 3 μM. The antiviral efficacy against HCV was further enhanced by combining parthenolide with interferon-α compared to interferon-α alone.^20^

Costunolide (33) has been extensively studied for its diverse pharmacological activities. Its structure features a 10-membered ring skeleton with a monocarboxylic acid and three double bonds. Initially isolated from the roots of *Saussurea lappa* Clarke, costunolide has emerged as a promising therapeutic candidate for the development of anti-HBV drugs. Research conducted by Chen *et al.*^14^ demonstrated that costunolide inhibited the production of HBsAg in human hepatoma Hep3B cells, with an IC_50_ value of 1.0 μM. Furthermore, at a concentration of 4 μM, costunolide significantly reduced the mRNA expression levels of HBsAg in these cells.

In addition to its effects on HBV, costunolide (33) has also exhibited antiviral activity against herpes simplex virus type 1 (HSV-1), achieving an antiviral activity percentage of 15.39 ± 4.83%. It demonstrated some effectiveness against hepatitis A virus (HAV) as well, with an antiviral activity percentage of 2.18 ± 3.1%.^18^ These findings underscore the potential of costunolide and other sesquiterpene lactones as valuable candidates in antiviral therapy.

11(13)-Dehydroivaxillin (34) has been isolated from *Carpesium abrotanoides* L. belonging to Asteraceae.^41^ It is to be noted that approximately fifty sesquiterpenes have been reported from this medicinal herb with several bioactivities including antifungal, antibacterial, antitumor, insecticidal and anti-inflammatory activities. Additionally, sesquiterpene lactones, such as dicarabrol, reported from *C. abrotanoides* L, revealed promising antiviral against H1N1 and H3N2 activity.^44^ In a study conducted Yu-Qing He and his team,^44^ 11(13)-dehydroivaxillin (34) showed strong anti-influenza A (H1N1) virus activity with an IC_50_ value of 11.6 μM. Consequently, this compound may serve as potential anti-influenza A (H1N1) virus therapeutic agent in the future. In another study, 11(13)-dehydroivaxillin (34) showed a strong anti-influenza A H1N1 and H3N2 viruses' activity with IC_50_ values of 10.8 and 11.6 μM.^22^

In the study by Park *et al.*,^25^ the germacranolide SL, Ixerin H (35), exhibited significant antiviral activity against both Coxsackievirus B3 (CVB3) and Echovirus 7 (EV7). The antiviral activity indices were measured at 33.73 ± 1.93% for CVB3 and 61.70 ± 1.12% for EV7. These results indicate that Ixerin H demonstrated the most consistent antiviral activity compared to other guaianolides (9–14) isolated from the roots of *Ixeris dentata*.

Zaluzanin C (36), one of the other three SLs reported by Moustafa *et al.*,^18^ demonstrated antiviral activity against herpes simplex virus type 1 (HSV-1) and hepatitis A virus (HAV). The inhibitory activity percentages were 79.93 ± 7.17% for HSV-1 and 53.7 ± 10.05% for HAV, indicating a notable effectiveness against both viruses.

### 3.3. Antiviral activities of eudesmanolide SLs

Alantolactone (37), a eudesmanolide sesquiterpene lactone derived from *Inula helenium*, has been reported to exhibit potent antiviral activity against Hepatitis C Virus (HCV), with an EC_50_ value of less than 3 μM.^20^ This compound is also found in several plants within the Asteraceae family, including *Ajania fruticulosa*, *Aucklandia lappa* (formerly *Saussurea lappa*), *Carpesium macrocephalum*, *Chrysanthemum indicum*, *Inula racemosa*, *Saussurea costus* (Falc.) Lipsch., and *Telekia speciosa* (Schreb.) Baumg.^45^

Santamarin (38), the fourth sesquiterpene lactone reported Moustafa *et al.*,^18^ exhibited antiviral activity against herpes simplex virus type 1 (HSV-1), and hepatitis A virus (HAV) with inhibitory activity percentages of 92.91 ± 2.47% and 42.71 ± 10.47%, respectively.

Additionally, artesunate (39) and artemisinin (40), found in *Artemisia annua*, have shown anti-HBV activity. This was assessed by measuring the release of surface protein (HBsAg) and HBV-DNA in the HepG2 2.2.15 cell line, resulting in IC_50_ values of 2.3 μM and 0.5 μM for artesunate, and 55 μM and >100 μM for artemisinin, respectively.^15,34^

In a related context, Sas *et al.*^46^ published on the potential of sesquiterpene lactone endoperoxides to treat hepatitis C infections, yellow fever, dengue fever, bovine viral diarrhoea and classical swine fever. This study included *in vitro* screening of artesunate (39), artemisinin (40), artemether (41) and dihydroartemisinin (42) against DNA-viruses, retroviruses and *Flaviviridae*. Results showed strong activity of artemisinin (40) against the bovine viral diarrhoea virus (BVDV) which shares similarities with hepatitis C virus (HCV). The authors concluded that endoperoxides, particularly artemisinin (40), exhibit significant efficacy as treatments for hepatitis C and other *Flaviviridae* viral infections.

### 3.4. Antiviral activities of eremophilanolide SLs

Three enantiomeric sesquiterpene lactones—saluenolide A (43a) and its two dimers (43b and 43c)—were isolated from *Senecio* species, a widely distributed Chinese medicinal herb traditionally used for treating hepatitis B. The anti-HBV activity of these purified compounds was assessed, revealing that all three exhibited suppressive effects on the expression of HBsAg and HBV e antigen (HBeAg) in the HepG2.2.15 cell line. Real-time quantitative PCR analysis demonstrated that the compounds reduced the number of infectious virions released; however, they did not inhibit intracellular HBV DNA levels. These findings suggest that enantiomeric sesquiterpene lactones may have the potential to work synergistically with other antiviral agents in the treatment of HBV infection.^37^

### 3.5. Antiviral activities of carabrane SLs

Dicarabrol (44) and carabrol (45) are carabrane sesquiterpenoids reported to have significant antiviral activity against influenza viruses, such as H1N1 and H3N2.^22^

### 3.6. Antiviral activities of xanthanolides SLs

This class of sesquiterpenoids, known as xanthanolides, is primarily reported from the genus *Xanthium* (family Asteraceae). Xanthanolides are structurally characterized by a γ-butyrolactone moiety attached to a seven-membered ring and can be classified into two main types: (a) *cis*-fused lactones (*e.g.*, 8-*epi*-xanthatin, based on xanthanolide numbering) and (b) *trans*-fused lactones. Four xanthanolides (compounds 46–49) have been identified in the aerial parts of young *Xanthium spinosum* L. plants. Among these, xanthatin (47) demonstrated broad-spectrum antiviral activity against a range of viruses, including herpes simplex, vaccinia, and vesicular stomatitis in HEL cell cultures; feline coronavirus and feline herpesvirus in CRFK cell cultures; and vesicular stomatitis virus, coxsackievirus B4, and respiratory syncytial virus in HeLa cell cultures. However, it showed no inhibitory effects against the tested strains of influenza viruses (influenza A H1N1, influenza A H3N2, and influenza B).^38^

Xanthatin (47) has been reported from several genera of *Xanthium* and other genera of the Asteraceae family using different organic solvents including acetone, dichloromethane, and methanol.^38^ Geissman and co-workers converted xanthinin to xanthatin by addition of sodium acetate in ethanol.^47^ The antiviral effect of xanthatin (47) was assessed toward herpes simplex virus type 1 (HSV-1); herpes simplex virus type 2 (HSV-2); varicella-zoster virus (VZV); vesicular stomatitis virus; vaccinia virus; feline corona virus (FIPV); feline herpes virus; coxsackie virus B4; respiratory syncytial virus; influenza A H_1_N_1_; influenza A H_3_N_2_; influenza B; parainfluenza-3 virus; reovirus-1; Sindbis virus and Punta Toro virus. The activity was tested based on the inhibition of virus induced cytopathicity or plaque formation in human embryonic lung (HEL) fibroblasts, African green monkey cells (VERO), human epithelial cells (HeLa) or in Crandell–Rees feline kidney cells (CRFK). Xanthatin (47) demonstrated inhibitory activity against several viruses including herpes simplex, vaccinia and vesicular stomatitis in HEL cell cultures, feline corona, feline herpes in CRFK cell cultures, vesicular stomatitis virus, coxsackie virus B4 and respiratory syncytial virus in HeLa cell cultures. Additionally, *xanthatin did not show inhibition activity against the tested three influenza* types (influenza A H_1_N_1_, influenza A H_3_N_2_ and influenza B). Moreover, xanthatin showed pronounced cytotoxic activity against MDCK cell cultures of influenza and also against Vero cell cultures with minimal cytotoxic concentrations (MCCs) of 4 and >20 μM, respectively.^38^

### 3.7. Unusual sesquiterpene lactones

Bioassay-guided fractionation of the ethanolic extract of *Illicium henryi* identified that the chloroform fraction exhibited significant inhibitory effects on the secretion of hepatitis B virus (HBV) surface antigen (HBsAg) (IC_50_ = 0.58 mg mL^−1^, SI = 3.8) and HBV e antigen (HBeAg) (IC_50_ = 0.58 mg mL^−1^, SI = 3.8) in HBV-infected 2.2.15 cells. Further phytochemical investigation of this active fraction led to the isolation of 15 sesquiterpene lactones. These sesquiterpene lactones (compounds 50–64) were tested for their anti-HBV activities, with the most potent compounds being tashironin (50) and tashironin A (51). Tashironin showed IC_50_ values of 0.48 mM (SI = 6.3) for inhibiting HBsAg secretion and 0.15 mM (SI = 20.1) for inhibiting HBeAg secretion, while tashironin A exhibited IC_50_ values of 0.49 mM (SI = 2.1) and 0.15 mM (SI = 6.7), respectively, in HBV-transfected HepG2.2.15 cells.^39^

Moreover, two more sesquiterpene lactones, tashironin (50) and tashironin A (51), were also isolated from the roots of another species, *Illicium verum* Hook. F., and demonstrated antiviral activity against HIV in C8166 cells with EC_50_ values of 44.0 and 41.8 μM and SI values of 5.4 and 6.2, respectively.^40^

## 4. Antiviral activities of some sesquiterpene lactones through *in silico* studies

**Fig. 4:**
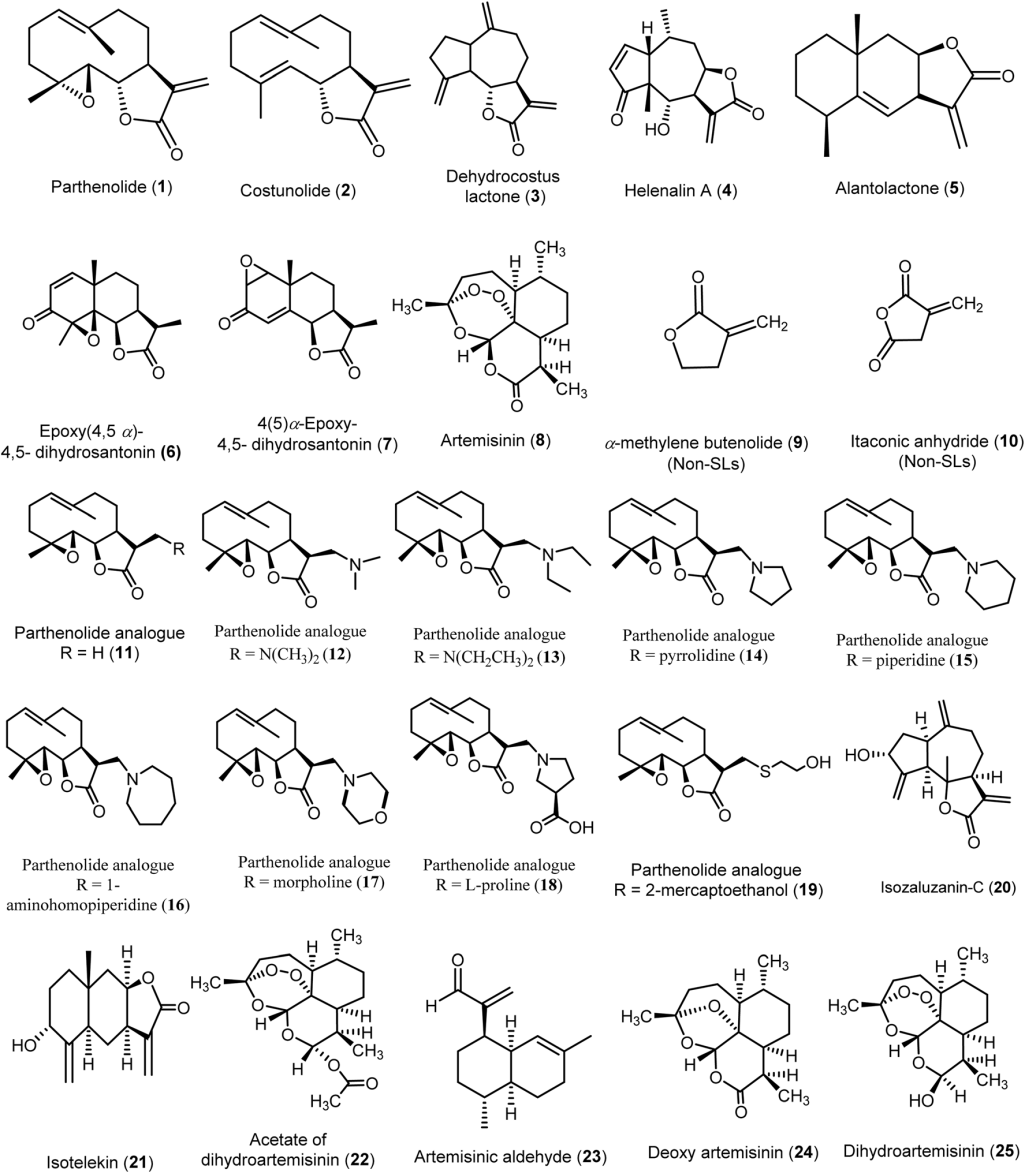
Chemical structures of compounds (1–25) evaluated using *in silico* models.

Several virtual screening studies were conducted on specific SLs (Fig. 4), including some of those mentioned above, to explore their potential as novel antiviral agents. These studies aimed to identify compounds with improved physicochemical and pharmacokinetic properties and to correlate the observed antiviral activities with *in silico* predictions. By doing so, researchers sought to elucidate structural requirements essential for antiviral efficacy, providing valuable insights for the development of new antiviral drugs.

A preliminary study was conducted to predict the quantitative relationship between seventeen sesquiterpene lactones (SLs 1–8 & 11–19) and two non-sesquiterpene lactone compounds (9–10) regarding their inhibitory activities against hepatitis C virus (HCV). The study utilized multiple linear regression (MLR) and self-organizing maps (SOM) as predictive modeling tools. By analyzing the chemical structures and classifying the compounds into active and inactive groups based on their chemical characteristics, the study provided valuable insights into key structural features influencing antiviral activity. These findings may contribute to refining the design of more effective HCV inhibitors.

The active compounds comprised various classes of sesquiterpene lactones (SLs), including germacranolides (1, 2, 12–18), a guaianolide (3), a pseudo-guaianolide (4), and an eudesmanolide (5). Notably, most eudesmanolides (6, 7, 8) displayed no activity. All active compounds with an EC_50_ of 10 μM or less possessed the *exo*-α-methylene-γ-lactone moiety, which appeared essential for anti-HCV activity, as seen in compounds (1–5) or their substituted analogs that retained this structural feature (11–18). Additionally, the 7β–8β configuration in the fusion of the α-methylene-γ-lactone ring with the terpenoid skeleton played a significant role in enhancing anti-HCV activity, as demonstrated by compound 4 (EC_50_ = 1.25 ± 0.35 μM) and compound 5 (EC_50_ = 2.03 ± 0.15 μM). Interestingly, compound 5 was the only eudesmanolide that exhibited anti-HCV activity among the analyzed eudesmanolides, indicating the positive impact of this configuration on eudesmanolides and pseudo-guaianolide derivatives. On the other hand, more symmetrical molecules with increased sphericality—and consequently, a higher influence of oxygen atoms—showed reduced biological activity. This was evident in compound 3 (EC_50_ = 3.08 ± 0.60 μM), which exhibited lower activity, and in the inactive compound 6 (EC_50_ > 10 μM), which contained more oxygen atoms compared to the active compound 3. Moreover, it was predicted that the presence of amino substituent groups at position 13 in most parthenolide analogs (12–18) contributed to anti-HCV activity similar to that of parthenolide, with compound 15 (EC_50_ = 1.64 ± 0.01 μM) displaying higher anti-HCV activity than parthenolide 1 itself (EC_50_ = 2.21 ± 0.15 μM). Overall, this study identified key structural requirements for anti-HCV activity, serving as a reference for virtual screening of SLs for their anti-HCV potential.^15–17^

An *in silico* study was conducted on the allylic alcohols isozaluzanin-C (20) and isotelekin (21), synthesized from the sesquiterpene lactone analogues dehydrocostus lactone and isoalantolactone derived from *Saussurea lappa* Clarke and *Inula racemosa*. The aim was to develop novel antiviral drugs with enhanced physicochemical and pharmacokinetic properties, alongside reduced side effects. Both compounds exhibited favorable characteristics, including good oral bioavailability, drug-likeness, lower toxicity, and high biocompatibility. Isotelekin (21) emerged as the most potent compound, achieving full-fitness scores of −981.61 and −797.16, binding energies of −1.766 and 5.50 kcal mol^−1^, and Δ*G* values of −6.47 and −5.554. In comparison, the market drug Umifenovir showed full-fitness scores of −1154.6 and −787.72, binding energies of 6.4038 and 21.14 kcal mol^−1^, and Δ*G* values of −7.936 and −5.977 against the anti-COVID-19 targets PDB: 6Y2E and the Indian mutant PDB: 3K7I. Isozaluzanin-C (20) also demonstrated notable antiviral activity with full-fitness scores of −972.72 and −786.36, binding energies of 2.603 and 8.995 kcal mol^−1^, and Δ*G* values of −6.543 and −5.702. This study highlighted that incorporating an –OH group at carbon 3 in 10 guaianolides and carbon 5 in alantolides significantly enhanced the antiviral efficacy of these compounds.^48^

Moreover, an *in silico* ADMET study was conducted to evaluate the interactions of artemisinin (8), acetate of dihydroartemisinin (22), artemisinic aldehyde (23), deoxy artemisinin (24), and dihydroartemisinin (25) with the SARS-CoV-2 main protease (M^pro^). The results indicated that artemisinin and its derivatives exhibited good oral absorption and a bioavailability score of 0.55. The tested compounds demonstrated strong binding to the M^pro^ active site, specifically to the Cys145 residue, with binding energies ranging from −5.2 to −8.1 kcal mol^−1^. Among these, the acetate of dihydroartemisinin (22) exhibited the highest binding score of −8.1 kcal mol^−1^, followed by artemisinin (8) at −7.2 kcal mol^−1^. Furthermore, artemisinin (8) and dihydroartemisinin (25) formed the highest number of conventional hydrogen bonds, comparable to chloroquine (the reference compound). Notably, all selected compounds showed better affinities than chloroquine, except for artemisinic aldehyde (23). These findings suggest potent binding of artemisinin and its derivatives to the SARS-CoV-2 M^pro^, along with the stability of the complex during 100 ns of molecular dynamics simulation, providing evidence for artemisinin (8) as a potential inhibitor of the M^pro^ proteolytic process, which is crucial for viral replication.^36^

## 5. Conclusion

Sesquiterpene lactones (SLs) represent a versatile class of bioactive compounds with significant potential in antiviral therapy. Their diverse structures, particularly the presence of α-methylene-γ-lactone and other electrophilic moieties, play a crucial role in their ability to interfere with viral processes such as replication and entry. Extensive research on guaianolides, germacranolides, and other SL subtypes has revealed promising antiviral activity against a range of viruses, including hepatitis C, influenza, and herpes simplex. As interest in natural products for drug development grows, sesquiterpene lactones continue to stand out as valuable scaffolds for the development of novel antiviral agents. However, further studies are necessary to fully understand their mechanisms of action, optimize their efficacy, and assess their safety in clinical applications. The ongoing exploration of SLs will likely yield important advancements in antiviral drug discovery, positioning these compounds as key candidates in the fight against viral infections.

## Data availability

The data supporting this article have been included in the main manuscript.

## Author contributions

Conceptualization: Yhiya Amen. All authors (Yhiya Amen, Gehad Abdelwahab, Ahmed A. Heraiz, Mahmoud Sallam, and Ahmed Othman) contributed to data collection, report analysis, and manuscript drafting. All authors reviewed, edited, and approved the final version of the manuscript.

## Conflicts of interest

The authors state no conflict of interest.
